# Profiling Peripheral Blood Lymphocytes in Alzheimer’s Disease and Mild Cognitive Impairment

**DOI:** 10.1002/alz.086090

**Published:** 2025-01-09

**Authors:** Yi Ling Clare Low, Jiaqi Sun, Xin Huang, Catarina F Almeida, Ben J Gu, Yoshiteru Kagawa, Colin L. Masters, Liang Jin, Yijun Pan

**Affiliations:** ^1^ Florey Institute of Neuroscience and Mental Health, Parkville, VIC Australia; ^2^ University of Melbourne, Parkville, VIC Australia; ^3^ Innate Phagocytosis Laboratory, Jumar Bioincubator, Melbourne, VIC Australia; ^4^ Peter Doherty Institute for Infection and Immunity, Parkville, VIC Australia; ^5^ Tohoku University, Sendai Japan; ^6^ Florey Institute of Neuroscience and Mental Health, University of Melbourne, Parkville, VIC Australia; ^7^ Monash University, Parkville, VIC Australia

## Abstract

**Background:**

In Alzheimer’s disease (AD), a major gap remains in the understanding of how the interplay between peripheral and central immune systems drives neuroinflammation and disease progression. More recently, the concept of brain lymph drainage has sparked interest as it may shed light on how the dynamics of T cell interactions contribute to AD. Our preliminary study aims to characterize alterations in the peripheral blood lymphocyte population among individuals with AD‐dementia and mild cognitive impairment (MCI), as compared with cognitively unimpaired (CU) individuals.

**Method:**

The peripheral blood lymphocyte population of 142 participants (AD‐dementia=34, MCI=47, CU=63) recruited from the Australian Imaging, Biomarkers and Lifestyle (AIBL) Study was profiled. Peripheral blood samples were collected for flow cytometry analysis. Cell surface markers facilitated the identification of various B and T cell populations including CD19^+^ pan B cells, CD4^+^ helper T cells, CD8^+^ cytotoxic T cells, CD4^+^CD25^+^CD127^‐^ regulatory T cells, CD4^+^CD8^+^ double positive T cells, and TCRγδ^+^ T cells, further categorized into subsets of CD4^‐^CD8^‐^, CD4^‐^CD8^+^ and CD4^+^CD8^‐^ TCRγδ^+^ T cells.

**Result:**

The study revealed a lower percentage of CD4^+^CD25^+^CD127^‐^ regulatory T cells in the AD‐dementia group compared to the CU group (P=0.034, AD=6.89±1.83, CU=8.27±2.21). Significant intergroup differences were identified in the CD4^+^CD8^+^ double positive T cell population (P=0.0363), with the MCI group displaying the highest levels (0.60±0.34), followed by the AD‐dementia (0.47±0.24) and CU group (0.42±0.23). Additionally, TCRγδ^+^ T cells were found to be elevated in the AD‐dementia group when compared with the CU group (P=0.0023, AD=15.4±14.4, CU=7.70±9.51). Intergroup variations were also observed in the subsets of CD4^+^CD8^‐^TCRgd^+^ T cells (P=0.0002) and CD4^‐^CD8^+^TCRgd^+^ T cells (P=0.0004). However, no significant differential changes were found between the groups for CD19^+^ pan B (P=0.24), CD4^+^ helper T (P=0.29), and CD8^+^ cytotoxic T (P=0.56) cell populations.

**Conclusion:**

This preliminary analysis highlights disease status‐associated changes in the peripheral blood lymphocyte profile, offering potential insights into the role of peripheral immunity in AD pathology. Further research targeting alterations in the peripheral blood lymphocyte profile is warranted to uncover novel biomarkers, enabling early detection or prediction of disease severity/progression of AD.

